# Coronary Assessment and Revascularization Before Transcutaneous Aortic Valve Implantation: An Update on Current Knowledge

**DOI:** 10.3389/fcvm.2021.654892

**Published:** 2021-05-21

**Authors:** Muhammad Sabbah, Thomas Engstrøm, Ole De Backer, Lars Søndergaard, Jacob Lønborg

**Affiliations:** Department of Cardiology, Rigshospitalet, Copenhagen University Hospital, Copenhagen, Denmark

**Keywords:** transcatheter aortic valve implantation, percutaneous coronary intervention, revascualrization, fractional flow reserve, coronary artery diasease

## Abstract

Transcutaneous aortic valve implantation (TAVI) has led to a paradigm shift in the treatment of severe aortic stenosis (AS) in the elderly and is expanding to still younger and lower-risk patients with severe AS as an alternative to surgical aortic valve replacement (SAVR). While the role of coronary artery bypass grafting with SAVR is well-documented, the analog of percutaneous coronary intervention with TAVI is less so. The aim of this review is to provide an overview of the important challenges in treating severe AS and co-existing coronary artery disease in patients planned for TAVI.

## Introduction

Aortic stenosis (AS) is the most common valvular heart disease in the Western world, affecting 2–7% of all people older than 65 years (1, 2). Surgical aortic valve replacement (SAVR), introduced in the 1960s (3), was for many years the only treatment available for severe AS, but excluded a considerable number of patients due to high surgical risk. The advent of transcatheter aortic valve replacement (TAVI) in 2002 has led to a paradigm shift in the treatment of severe AS (4). TAVI has been demonstrated to confer better survival compared with conservative treatment in inoperable patients (5–8). Moreover, it has been shown to be at least non-inferior to SAVR in elderly patients across all surgical risk profiles (9–15). According to the recently updated American and European guidelines, TAVI is the recommended treatment of symptomatic severe AS in patients aged 80 years or more and may be considered in patients aged 65–80 years based on patient/anatomical characteristics and shared decision-making (16, 17). As a result, more patients are currently treated with TAVI than with SAVR in the Western world. In parallel with this increasing number of (now also younger) patients treated with TAVI, there is an increasing focus on dealing with co-existing coronary artery disease (CAD).

### Prevalence, Importance, and Challenges of Coronary Artery Disease in Patients With AS

AS and CAD share several common cardiovascular risk factors such age, hypertension, hypercholesterolemia, and smoking (18). Likewise, there is an important overlap in the symptomatology of AS and CAD with exertional dyspnea and angina pectoris seen in both. Frequently, no angina is reported in patients with severe AS, but significant CAD is incidentally found in the coronary angiogram. In other cases, patients report classical angina but have no significant CAD, the cause likely being microvascular dysfunction (19). Thus, the relative contribution from each disease to the symptom burden is often hard to discern. The prevalence of CAD in patients undergoing TAVI is reported to range from 38.0 to 74.9% (5–7, 12, 13, 20–31). This broad range reflects a large variation in the definition of CAD between studies (Table 1). However, as both previous coronary artery bypass graft (CABG) and percutaneous coronary intervention (PCI) are included in the definition of CAD in these studies as well as the coronary stenosis visually on the coronary angiogram without physiology to assess the importance of the CAD has been used to define co-existing CAD, the prevalence of significant CAD that may warrant revascularization in addition to TAVI is likely much lower. This is supported by registry data in which only 15% of a TAVI population underwent revascularization with PCI before TAVI (24).

**Table 1:** Prevalence, definition, and importance of CAD in TAVI reported in randomized trials and real-world multi-center registries.

**Study**	**Year**	**Sample size (*n*)**	**CAD (%)**	**Definition of CAD**	**Mean age**	**STS score**	**Logistic EuroSCORE**	**Prognostic importance of CAD**
**Randomized trials**								
PARTNER 1 (5)	2011	348	74.9	Not specified	83.6 ± 6.8	11.8 ± 3.3	29.3 ± 16.5	–
COREVALVE (6)	2014	390	75.4	Not specified	83.2 ± 7.1	7.3 ± 3.0	17.6 ± 13.0	–
PARTNER 2 (12)	2016	1,011	69.2	Not specified	81.5 ± 6.7	5.8 ± 2.1	–	–
SURTAVI (13)	2017	864	62.6	Not specified	79.9 ± 6.2	4.4 ± 1.5	11.9 ± 7.6	–
**Multi-center registries**								
SOURCE (30)	2011	1,038	51.7	Not specified	81.7 ± 6.7^*^	–	25.8 ± 14.4^*^	CAD not associated with increased 1-year mortality in multivariable analysis
FRANCE 2 (20)	2012	3,195	47.9	Not specified	82.7 ± 7.2	14.4 ± 11.9	21.9 ± 14.3	CAD not associated with increased 1-year mortality
German TAVI registry (31)	2012	1,382	62.2	Not specified	81.5 ± 6.1^**^ 82.1 ± 6.3^***^	-	23.0 ± 14.6^**^ 16.4 ± 10.7^***^	CAD was associated with increased in-hospital mortality (OR 1.90, *p* < 0.01) but not in a multivariable logistic regression analysis (OR 1.40, *p* = 0.18)
Italian COREVALVE registry (20)	2013	659	38	PCI or CABG prior to TAVI	81.2 ± 5.8	–	23.1 ± 13.7%	CAD not associated with increased risk of 1-year mortality or MACCE. Complete revascularization was not associated with worse MACCE incidence compared with untreated patients
ADVANCE (21)	2014	1,015	57.8	Not specified	81.1 ± 6.4	–	16.0 (10.3, 25.3)	CAD did not predict 1-year mortality in a univariable model, HR 1.25, *p* = 0.159
German aortic valve registry (28)	2014	3,875	54.4	Not specified	81.1 ± 6.2	–	–	–
SOURCE-XT (22)	2015	2,688	44.2	Not specified	81.4 ± 6.6	7.9 ± 6.6	20.4 ± 12.4	CAD not associated with increased mortality in a multivariable analysis, HR 1.22, *p* = 0.055
UK TAVI registry (24)	2015	2,588	45.2	Stenosis >50% of luminal diameter of the left main stem or the three main coronary arteries or their major epicardial branches as demonstrated in the angiogram	81.31 ± 7.57	–	18.06 (12.08, 28.11)	CAD not associated with mortality at 4 years in a multivariable analysis, HR 1.14, *p* = 0.10
STS/ACC TVT Registry (23)	2016	26,414	63.1	Not specified	82	–	–	–
Singh (25)	2016	22,344	66.9	Not specified	81.2 ± 0.13	–	–	In-hospital mortality was higher for patients undergoing TAVI + PCI compared with TAVI alone in a propensity-matched multivariate logistic regression model (10.2 vs. 6.8%, *p* = 0.008). Higher rates of iatrogenic vascular, cardiac, respiratory, infectious complications in the TAVI + PCI group (*p* < 0.001 for all)
SOURCE 3 registry (26)	2017	1,947	51.5	Not specified	81.7 ± 6.7^*^	–	17.8 ± 12.9	–

*Data are presented means ± SD or as medians with interquartile range. CABG indicates coronary artery bypass graft; CAD, coronary artery disease; HR, hazard ratio; MACCE, major adverse cardiovascular and cerebrovascular events; MI, myocardial infarction; PCI, percutaneous coronary intervention; STS score, Society of Thoracic Surgeons score*.

* *Transfemoral TAVI*.

** *Patients with CAD*.

*** *Patients without CAD*.

Regardless, co-existing CAD is frequent in patients with severe AS undergoing TAVI, but the clinical importance is uncertain (Table 1). Registry data suggest that co-existing CAD is not independently related to a reduced 1-year survival rate (24). However, these data may be confounded as a substantial number of patients underwent revascularization with percutaneous coronary intervention (PCI) before TAVI. Moreover, the definition of CAD was based solely on visual assessment of the coronary angiogram. Today, it is well-known that angiography by itself is an inaccurate method for evaluation of the physiological severity of coronary stenoses (32). Instead, fractional flow reserve (FFR)–guided revascularization is the gold standard (33–36). Data from another registry using SYNTAX score for evaluation of CAD indicate that patients with severe and anatomically complex CAD undergoing TAVI have an increased cardiovascular mortality compared with patients with no or mild to moderate CAD (37). In the most recent meta-analysis addressing the impact of CAD in patients undergoing TAVI, 15 non-randomized studies were included for analysis (nine studies were retrospective and six prospective) totaling more than 5,000 patients (38). The main findings were as follows: (1) 30-day all-cause mortality was similar for patients with and without CAD, but 1-year mortality was significantly higher in patients with CAD; (2) procedural complications such as myocardial infarction (MI), cardiovascular mortality, stroke, bleeding, and vascular complications were not different between groups. Conversely, recently published data show that almost 10% of patients treated with TAVI are readmitted with acute coronary syndrome after a median of 25 months, which is related to the presence of CAD (39). However, these results should be interpreted with caution due to heterogeneity in the definition of CAD, lack of physiological assessment of CAD severity or use of SYNTAX score, incomplete reporting of endpoints based on CAD status in some studies, as well as the observational nature of these studies which rules out assessment of causality between CAD and outcome. Moreover, as the patients selected for TAVI are getting younger with a longer life expectancy, the clinical importance of CAD may also change.

### Non-invasive Evaluation of CAD

Exercise and dipyridamole stress echocardiography have a high sensitivity for CAD in patients with AS but a specificity of only 61–74% with thallium-201 scintigraphy or coronary angiography as reference (40, 41). When adenosine is used as stressor, specificity is higher at 97% with a sensitivity of 85% (42). Other modalities such as stress SPECT, PET, and cardiac MR have been tested in small patient series (*n* = 23–50) with reported sensitivity ranging from 91 to 100% with specificity of 80–91% when compared against coronary angiography (43, 44). The utility of coronary CT angiography (CTA) has also been investigated in patients planned for TAVI (45, 46). In one registry, CTA done before TAVI identified significant CAD in 93.3% of patients who underwent PCI (46). However, the definition of significant CAD was a luminal narrowing of ≥50%, and only 10% had FFR measured. Another study reported CTA to have a negative predictive value of 96% for detection of significant CAD (45). Again, significant CAD was defined as ≥50% luminal narrowing, and the use of FFR was not reported. These data confirm that CTA is nearly as good as coronary angiography at identifying anatomical characteristics of CAD. Although, CTA by itself is not enough for accurate assessment of the functional significance of CAD, especially in intermediate coronary stenoses, it does offer a high negative predictive value, which can spare some patients the risks of invasive testing. As TAVI is moving toward still younger patients—with a lower prevalence of CAD—CTA could contribute to better cost-effectiveness. The accuracy of CTA can be improved further by post-processing using computational fluid dynamics which allows for derivation of FFR non-invasively (FFR_CT_) (47). The CAST-FFR study evaluated FFR_CT_ against invasive FFR in patients with severe AS (48) and reported better accuracy than CTA alone. Unfortunately, clinical implementation of FFR_CT_ has been limited by its dependence on the quality of source data, added cost, and requirement for time-consuming computation (49).

### Invasive Evaluation of CAD

#### Fractional Flow Reserve

**Figure 1:**
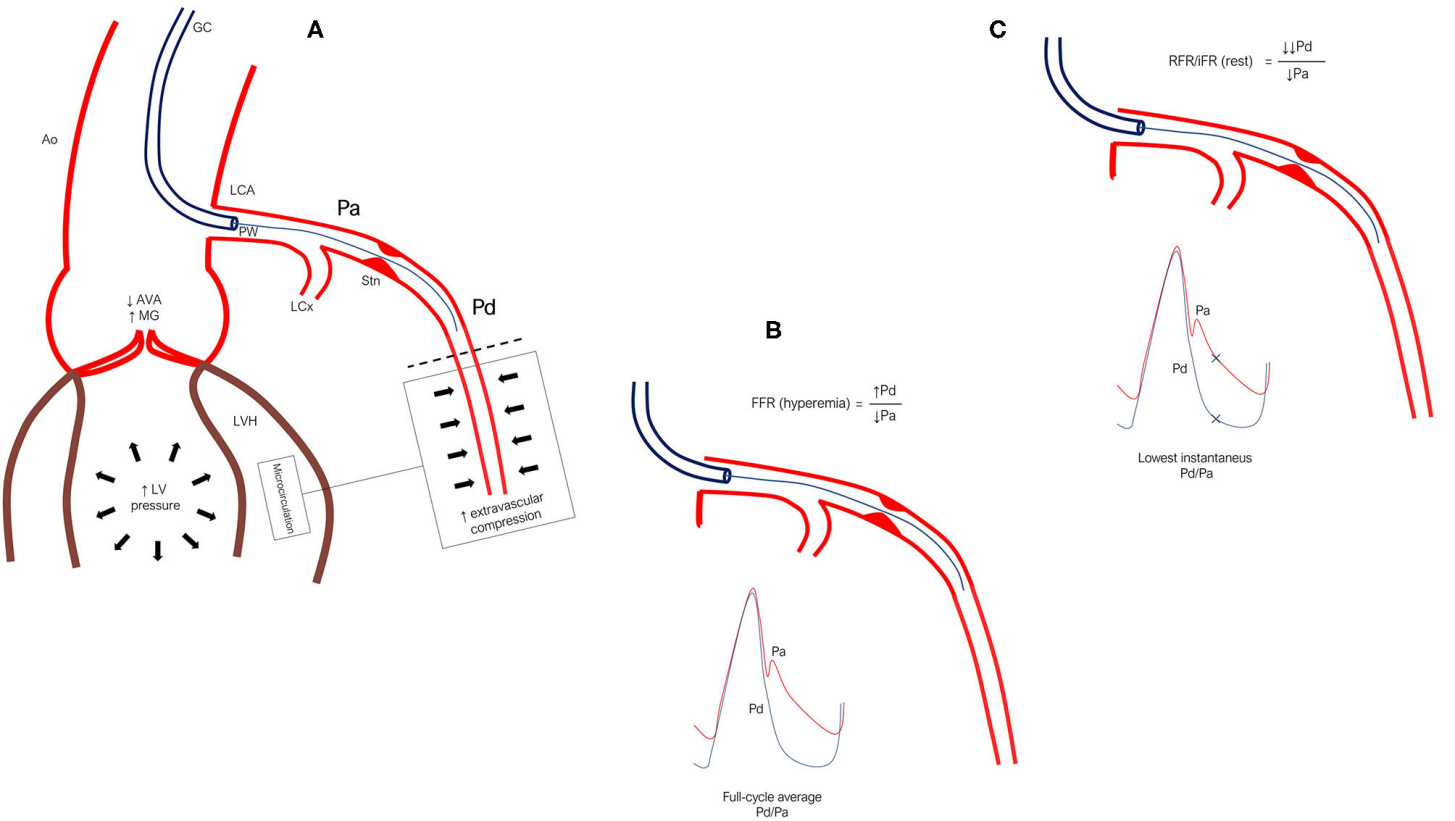
**(A)** In severe aortic stenosis, systemic, and thus aortic pressure, Pa, is often reduced due to pressure loss across the aortic valve. Meanwhile, elevated LV pressure and increased contraction force due to LVH causes intramyocardial compression of the microcirculation, driving up mean distal coronary pressure, Pd (back-pressure). **(B)** FFR is measured as the ratio of full-cycle mean Pd/Pa during maximal hyperemia. As such, flow rate and subsequent pressure loss across the epicardial stenosis would not be expected to vary much before vs. after TAVI. However, relief from extravascular compression (LVH and high LV pressure especially during systole) after TAVI may cause Pd to fall, thus lowering FFR. **(C)** The major difference between FFR and iFR/RFR is that the latter are measured during rest and are calculated as the lowest instantaneous Pd/Pa, which typically occurs during diastole. This is a potential source of error in severe AS because resting flow rate is elevated due to increased myocardial workload. Therefore, when pressure is sampled only in diastole—where pressure separation is very large in AS patients—the calculated Pd/Pa can turn out to be lower than that calculated from the full-cycle averages of Pd/Pa even during maximal hyperemia (i.e., FFR). As pressure loss across a stenosis is closely related to flow rate, measuring IFR/RFR before TAVI likely overestimates the significance of a stenosis as compared with evaluation after TAVI where resting flow and subsequent pressure loss are drastically reduced. Ao, indicates aorta; AVA, aortic valve area; FFR, fractional flow reserve; GC, guide catheter; iFR, instantaneous wave-free ratio; LCA, left coronary artery; LCx, left circumflex artery; LV, left ventricle; LVH, left ventricular hypertrophy; MG, mean gradient; Pa, aortic pressure; Pd, distal coronary pressure; PW, pressure wire; RFR, resting full cycle ratio; Stn, stenosis.

The use of physiological testing in addition to standard coronary angiography ensures the most accurate assessment of CAD severity (34). Of the currently available pressure-derived indices of stenosis severity, FFR is the only one that has been validated against a true gold standard (prospective multi-testing Bayesian methodology) (50) and is therefore the gold standard for invasive assessment in patients without AS. Although, FFR has never been validated in patients with severe AS, registry data indicate that FFR may also be applicable in patients with AS (51). FFR appears to change only slightly when measured immediately after TAVI (52). Positive FFR values tend to worsen, whereas, negative values tend to improve. In 6% (8 out of 133 lesions), FFR crossed the threshold for treatment indication (52). However, as others have pointed out, the prevailing systemic hemodynamic conditions during TAVI call for caution in interpretation of FFR values measured peri-procedurally (53). Physiologically, the main factor thought to alter FFR in AS patients is increased microvascular resistance due to the extravascular compression caused by high ventricular pressures (Figure 1). In a small observational study, FFR was measured in 23 lesions all with FFR values >0.75 (14 patients) before TAVI and 14 months later (54). Abnormally low values tended to worsen at follow-up while lesions with a FFR >0.80 remained stable. Only one lesion crossed the ≤0.80 cut-off from baseline to follow-up. However, this study only included patients with FFR >0.75 and not the full spectrum of FFR values. The bottom line, however, is that the standard FFR cut-off of ≤0.80 in severe patients with AS is unproven. Likewise, it is unknown if deferral of PCI in lesions with FFR > 0.80 is safe in this patient group. The ongoing FAITAVI trial (ClinicalTrials.gov Identifier: NCT03360591) is designed to address some of these questions. It is planned to randomize 320 patients referred to TAVI to revascularization guided by either angiography or FFR.

#### Resting Indices

Adenosine infusion is considered safe (55) and causes no significant change in cardiac work (56). However, adenosine frequently causes systemic hypotension, chest pain, and shortness of breath. These adverse effects can be an issue in frail patients hemodynamically challenged by severe AS and CAD. Efforts to circumvent the use of adenosine lead to the development of the resting full-cycle ratio (RFR) and instantaneous wave-free ratio (iFR). Resting indices are appealing as they offer a simpler, cheaper, and faster stenosis evaluation without the adverse effects of adenosine. However, they have an important potential limitation when used in patients with AS. In severe AS, resting myocardial workload is increased due to increased afterload (57). Accordingly, resting myocardial blood flow and subsequent trans-stenotic pressure-drop are large, falsely decreasing RFR/iFR (Figure 1). After valve replacement, afterload is abruptly reduced and left ventricular hypertrophy gradually regresses (58). Consequently, resting flow and trans-stenotic pressure-drop must decrease. It thus follows that the appropriate time for stenosis evaluation may be after TAVI. Using a resting flow index before TAVI, one may probe a physiologically significant lesion which after valve replacement becomes non-significant. In the only published study reporting long-term changes in FFR and iFR (14 months after TAVI), iFR showed a higher reclassification rate at 21.7 vs. 4.3% for FFR (54). Reclassification was due to lesions becoming non-significant after TAVI. Published data on changes in FFR and iFR before and after TAVI are summarized in Table 2.

**Table 2:** FFR and iFR measured before and right after TAVI and after 14-month follow-up.

		**Number of lesions**	**Pre-TAVI**	**Post-TAVI**	**References**
**FFR before and right after TAVI**					
LAD	FFR ≤ 0.80	15	0.72 ± 0.12	0.69 ± 0.13	Pesarini et al. (52)
	FFR > 0.80	41	0.88 ± 0.12	0.89 ± 0.13	
Other than LAD	FFR ≤ 0.80	6	0.69 ± 0.12	0.62 ± 0.14	
	FFR > 0.80	71	0.94 ± 0.12	0.95 ± 0.13	
Reclassification rate				FFR	
				8/133 (6%)	
FFR, all vessels		23	0.87 (0.85–0.92)	0.88 (0.83–0.92)	Scarsini et al. (54)
iFR, all vessels		23	0.88 (0.85–0.96)	0.90 (0.83–0.93)	
**FFR/iFR before TAVI and at 14-month follow-up**					
FFR, all vessels		23	0.87 (0.85–0.92)	0.88 (0.82–0.92)	Scarsini et al. (54)
iFR, all vessels		23	0.88 (0.85–0.96)	0.91(0.86–0.97)	
Reclassification rate			iFR	FFR	
			7/23 (21.7%)	1/23 (4.3%)	

*Data are presented as mean ± SD or median with interquartile range. Reclassification rates of FFR and iFR values in Scarsini et al. were only reported at 14-month follow-up. FFR indicates fractional flow reserve; iFR, instantaneous wave-free ratio*.

### Revascularization in Patients Treated With TAVI

There are several uncertainties regarding revascularization of CAD before TAVI: (1) Should patients selected for TAVI with significant CAD undergo PCI?; (2) the optimal order in which to do PCI and TAVI is unknown; (3) consequences of PCI on anti-thrombotic therapy; (4) the choice of treatment for patients with more complex CAD (complex PCI + TAVI or CABG + SAVR).

#### Is Revascularization Necessary?

Revascularization may provide symptom relief and prevent future events such as acute coronary syndrome, as has been demonstrated in patients with stable CAD without AS and in the treatment of non-culprit lesions in patients with STEMI (59–64). Revascularization may also help to hemodynamically stabilize the patients during TAVI, as discussed later. The obvious downside of PCI before TAVI is the necessary temporary treatment with dual antiplatelet therapy (DAPT) which increases bleeding risk, particularly in the elderly, that is, in patients currently undergoing TAVI. Even short-term DAPT is associated with a higher rate of serious adverse events as compared with single antiplatelet therapy, which is now the standard post-procedural anti-thrombotic therapy after TAVI (65, 66). Another concern regarding PCI in patients with AS is the risk of stent-thrombosis and target lesion failure, but recent data have shown that these events are rare in patients treated with PCI before TAVI (67). The scarcity of controlled data from patients with AS and CAD leaves one to rely on data from isolated CAD (68–72), from which may be extrapolated that factors such as left main stenosis, very proximal stenoses, and multi-vessel disease should mandate revascularization at *some point*, be it before or after TAVI. For example, there is little doubt that physiologically significant left main lesions are certainly important to treat. Conversely, very distal stenoses with a small downstream subtended myocardial mass and FFR values in the gray zone, i.e., 0.75–0.80, might not be worth the risks of PCI neither before nor after TAVI. Because patients with severe AS are typically old, one may argue that the combined prognostic impact of age itself and severity of AS significantly outweighs that of co-existing CAD, making the benefit of revascularization increasingly irrelevant. For example, in the PARTNER 2 trial, the event rate (death + stroke) at 2-year follow-up was 20% while the event rate (death + myocardial infarction) at 5-year follow-up in the FAME-2 trial was only 8% (12, 73). In a retrospective analysis of the DANAMI-3-PRIMULTI study, increasing age, ≥75 years, diminished the prognostic benefit of revascularization of non-culprit arteries in patients with STEMI (68). On the other hand, the After 80 trial showed a benefit of revascularization vs. medical treatment of NSTEMI or unstable angina in patients ≥80 years (74). However, that trial also found that increasing age diminished this benefit.

The central question of whether PCI before TAVI is beneficial or even necessary at all was addressed in the recently completed, but not yet published, ACTIVATION trial (75). They randomized patients with severe AS and at least one coronary stenosis >70% in a major epicardial coronary artery to either PCI or medical therapy before TAVI. CAD was evaluated by angiography only, and patients in CCS class III–IV were excluded. Unfortunately, the trial was stopped prematurely due to low enrollment rate, with only 235 out of planned 310 patients included. The rate of the primary endpoint of mortality and rehospitalization was 41.5% in patients treated with PCI and 44.0% in the control group, which did not meet the non-inferiority margin. Patients treated with PCI had more bleeding events (44.5 vs. 28.4%, *p* = 0.021) with no statistical difference in major bleedings (26.1 vs. 18.1%, *p* = 0.19). However, the trial did not include myocardial infarction and urgent revascularization in the primary endpoint—outcomes which arguably are more relevant in this patient group than all-cause mortality. Also, information on effect on symptom relief is warranted. Another concern is the use of angiography to guide treatment in the ACTIVATION trial, as FFR is the most optimal method to guide revascularization in patients without AS (34) and probably also in patients with AS (51). Although ACTIVATION provided important evidence, the role of revascularization and especially FFR-guided PCI in patients with severe AS is still unresolved. The ongoing NOTION-3 trial (ClinicalTrials.gov Identifier: NCT03058627) may provide additional information; it is planned to randomize a total of 452 patients with severe AS and CAD to either FFR-guided full revascularization before TAVI in a staged approach or TAVI alone. Primary endpoints are all-cause mortality, MI, or urgent revascularization until the last included patient has been followed for a year after the TAVI. Another trial, COMPLETE TAVR (ClinicalTrials.gov Identifier: NCT04634240), will randomize 4,000 patients referred for TAVI to either angiography-guided PCI after TAVI or medical treatment.

#### When Is the Optimal Time for Revascularization?

**Figure 2:**
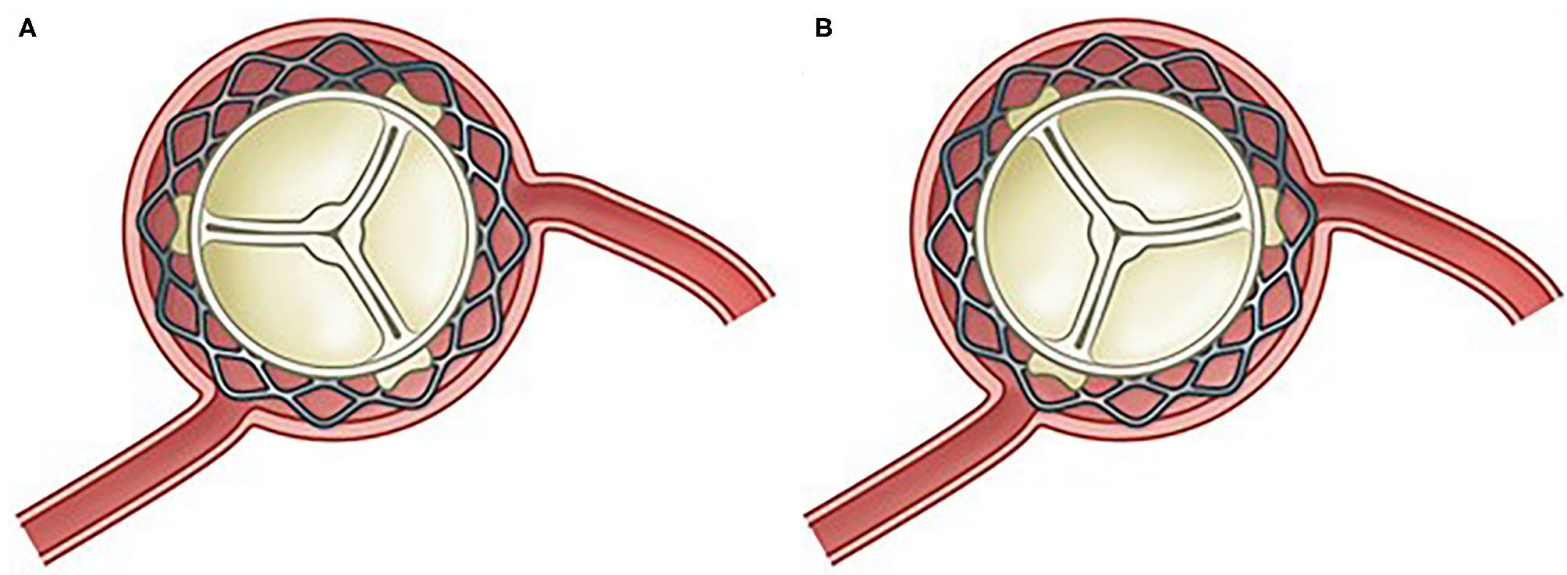
Commissural alignment between native and TAVI valve makes for easy coronary access **(A)** compared with commissural misalignment **(B)**.

**Figure 3:**
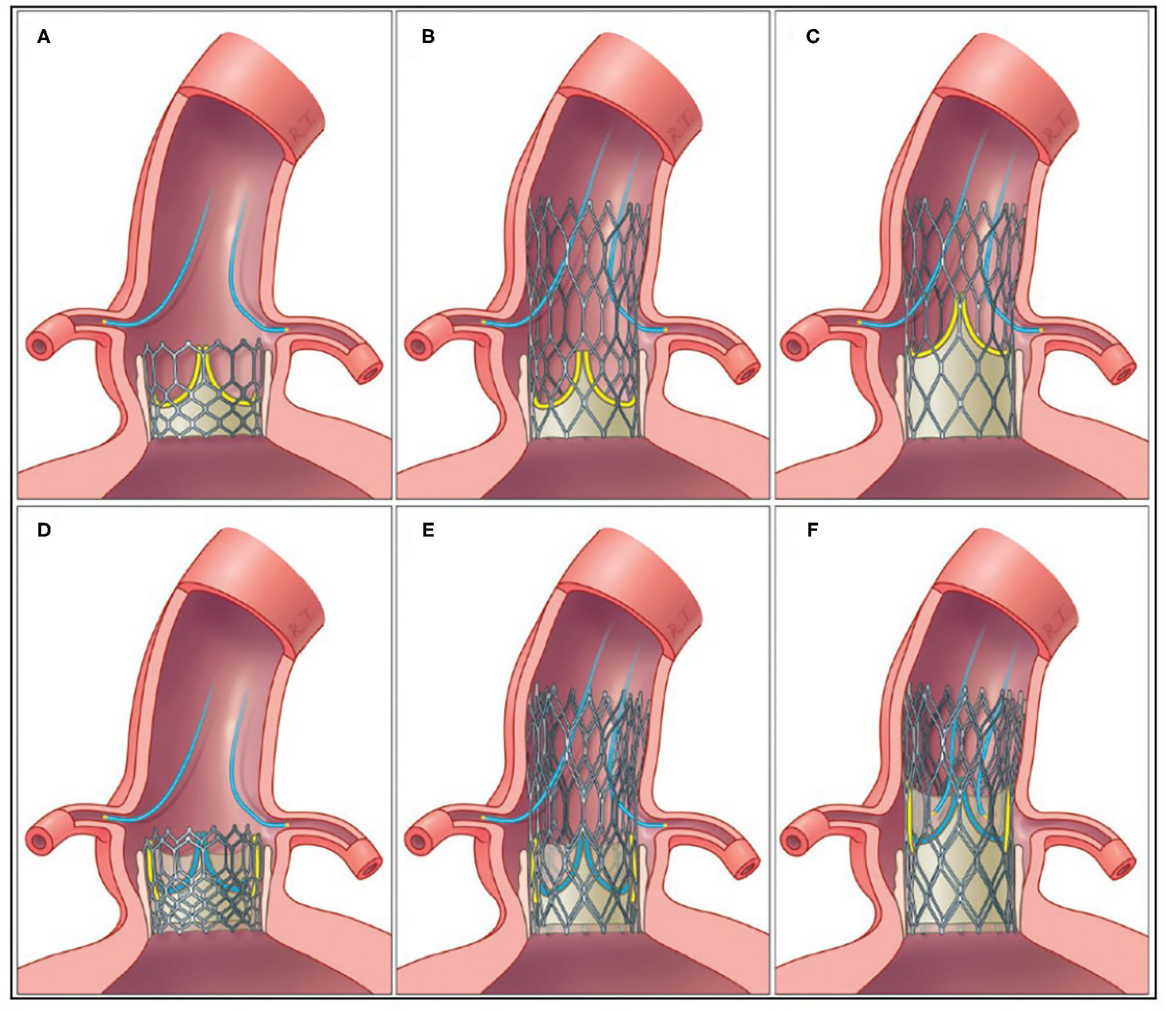
Coronary access after first TAVI with low-frame and intra-annular leaflet position **(A)**, high-frame and intra-annular leaflet position **(B)**, and high-frame and supra-annular leaflet position **(C)**. After TAVI-in-TAVI, access to the coronary arteries may be possible in patients with low-frame and intra-annular leaflet position **(D)** and high-frame and intra-annular leaflet position **(E)**, whereas, access may be compromised in high-frame and supra-annular leaflet position **(F)**. Yellow leaflets = leaflets in the first implanted THV; blue leaflets = leaflets in the second implanted THV; yellow/gray shading = tissue tunnel.

Revascularization done before, or in conjunction with TAVI, may help to avoid myocardial ischemia related to hemodynamic instability during the TAVI procedure, but experience with the TAVI procedure has shown us that this issue is a lesser concern (Figure 3). Another issue is the easier coronary access before compared with after TAVI (76). Coronary access after TAVI is subject to growing concern in patients with long life expectancy and therefore a higher risk of a second TAVI (valve-in-valve). Commissural alignment of the transcatheter heart valve may help in overcoming this issue (77), just as use of a transcatheter heart valve with a low frame and intra-annular leaflets allows easier coronary access as demonstrated in the RE-ACCESS study (76) (Figures 2, 3). There are no randomized data demonstrating whether concomitant TAVI and PCI is superior to a staged approach, or vice versa. In the only published meta-analysis including four observational studies with a total of 209 patients, there was no difference between groups in terms of 30-day mortality, renal failure, periprocedural MI, life-threatening bleeding, or major stroke (78). In the SURTAVI trial, 128 patients underwent TAVI and PCI of whom 76 (56.4%) were treated through a staged approach, whereas 52 (40.6%) had TAVI and PCI performed concomitantly. The staged approach was associated with significantly higher contrast load and acute kidney injury compared with the concomitant procedure (79). Although, patients were not randomized to either approach, this sample is the largest from a single published study. Contrarily, in patients with complex CAD and reduced left ventricular function, TAVI is generally recommended before revascularization. These patients, in turn, are disadvantaged by the dependence on the blood supply from a compromised coronary circulation during the TAVI procedure. The optimal order in which to do PCI and TAVI is currently under investigation in the TAVI-PCI Trial (ClinicalTrials.gov Identifier: NCT04310046) in which patients will be randomized to FFR-guided PCI before or after TAVI.

#### How to Revascularize Patients Undergoing TAVI?

Revascularization with PCI vs. CABG is another field in cardiovascular medicine of complexity that has gained much attention. In patients with AS and significant CAD the decision of performing CABG + SAVR or TAVI + PCI is even more complex. In the patients already selected for TAVI, percutaneous revascularization with PCI is undeniably the method of choice for revascularization. However, in patients without AS, CABG is preferred over PCI in patients with left main stenosis, three-vessel disease, and SYNTAX score >22, multivessel disease and diabetes or reduced LV function. Thus, as the complexity of CAD increases, the beneficial effect of PCI may be counterbalanced by increasing risk of complications and CABG + SAVR may be superior to PCI + TAVI in these patients. A recent meta-analysis comparing SAVR + CABG vs. TAVI + PCI found only three eligible studies out of 425 screened references (80). Of these, only one study was a randomized trial (79). The meta-analysis found no differences in 30-day safety outcomes (MI, stroke) and 2-year mortality. However, the authors reported differences in revascularization strategies, inaccuracies in surgical risk assessment, and non-uniformity in CAD grading according to SYNTAX score between studies (80). Nevertheless, this evidence suggests that TAVI + PCI is comparable with SAVR + CABG.

Taken together, patients with AS and co-existing CAD are heterogeneous in terms of risk profiles, comorbidities, life expectancy, severity of AS, and CAD as well as symptom burden. Importantly, both the functional severity of CAD (extent of coronary ischemia) and anatomical complexity (SYNTAX score) are highly variable and may both impact on the optimal of treatment and decision-making. Thus, Heart Team decisions focusing on individual patient–orientated treatment are important with contributions from invasive (coronary and structural) and non-invasive cardiologists as well as thoracic surgeons. Future studies may also help in addressing some of these pivotal questions in current Cardiology.

#### Anti-thrombotic Treatment After PCI?

DAPT is no longer recommended after TAVI as bleeding rates are higher without clear benefits (65, 66). However, TAVI patients who undergo PCI cannot avoid DAPT without increasing the risk of stent thrombosis in exchange for the lower bleeding risk. Moreover, longer DAPT treatment is especially recommended in the case of complex PCI, e.g., of bifurcations or venous grafts. In addition, more than one third of TAVI patients have concomitant atrial fibrillation with an indication for oral anticoagulation therapy (81). As such, the typically frail TAVI patient with atrial fibrillation and a need for complex PCI is exposed to an increased bleeding risk.

## Conclusions

TAVI has revolutionized the treatment of severe AS and the indication for TAVI is expanding to still younger and lower-risk patient groups. Important unresolved questions are if, how, and when to treat co-existing CAD. To date, data on these pivotal questions are few, but ongoing clinical trials are greatly awaited and will provide important evidence.

## Author Contributions

All authors have read and approved the manuscript and agreed to be accountable for all aspects of the work in ensuring that questions related to the accuracy or integrity of any part of the work are appropriately investigated and resolved.

## Conflict of Interest

The authors declare that the research was conducted in the absence of any commercial or financial relationships that could be construed as a potential conflict of interest.
